# Reviewed article: Oliveira TM, Araújo ACM, Costa GAT, Gomes LP, Alcantara TMPDC, Carvalho NS, Gusmão JD, Levi M, Burian APN, Ferraz ML, Silva TPR, Matozinhos FP. Social environment and services at the Special Immunobiological Reference Centers: epidemiological study, Minas Gerais, 2022-2024. Epidemiol Serv Saude. 2026:35;e20250092

**DOI:** 10.1590/S2237-96222026v35e20250092.b

**Published:** 2026-02-06

**Authors:** Mariana Del Grossi Moura

**Affiliations:** 1 Instituto Sírio-Libanês de Ensino e Pesquisa, São Paulo , SP, Brazil

## First round comments

Prezado(a) autor(a),

Agradeço a submissão do seu manuscrito intitulado “Ambiente social e atendimentos do Centro de Referência para Imunobiológicos Especiais de Minas Gerais, Brasil” e reconheço a relevância do tema abordado. Seu estudo traz uma contribuição valiosa, mas algumas melhorias são necessárias para aprimorar a clareza da apresentação, a solidez metodológica e a conformidade com as diretrizes da revista.

É fundamental que as sugestões e orientações dos revisores sejam analisadas com atenção, pois contribuirão significativamente para o aprimoramento do trabalho. A introdução pode ser mais objetiva, reduzindo trechos extensos de revisão da literatura e enfatizando com mais clareza a justificativa do estudo.

Para embasar melhor o delineamento metodológico, recomendo a consulta a artigos que discutem boas práticas na condução e relato de estudos ecológicos. Além disso, a escolha dos métodos estatísticos, especialmente o modelo logístico, precisa ser mais bem justificada. Também sugiro uma apresentação mais linear dos resultados, diferenciando com clareza as solicitações de imunobiológicos das prescrições efetivamente realizadas. Na discussão, algumas seções podem ser reorganizadas para evitar repetições e tornar a interpretação dos achados mais clara e objetiva.

Outro ponto essencial é a adequação do manuscrito às normas da revista, incluindo a revisão das referências para garantir conformidade com o estilo Vancouver, ajustes na estrutura das tabelas e adequação das palavras-chave ao DeCS.

Reitero a importância dessas adequações para que o manuscrito possa avançar no processo editorial. Aguardamos a versão revisada e nos colocamos à disposição para quaisquer esclarecimentos adicionais.

Recommendation: Major revision

## Second round comments

Prezados

Agradeço a submissão do seu manuscrito RESS-2025-0092.R1 intitulado “Ambiente social e atendimentos do Centro de Referência para Imunobiológicos Especiais de Minas Gerais, Brasil” e reconheço a relevância do tema abordado.

Após a revisão do seu artigo, identifico a necessidade de uma revisões menor (Minor Revision) para que o manuscrito esteja de acordo com os padrões editoriais da revista e para viabilizar sua publicação.

1. Recomendamos que a seção de Discussão seja reorganizada de forma a separar claramente a Conclusão do corpo da discussão, conforme a estrutura usual de artigos científicos. A criação de uma seção final intitulada “Conclusão” permitirá uma síntese mais objetiva dos principais achados e de suas implicações para a prática e para a formulação de políticas públicas.

2. A seção “Discussão” ainda se apresenta acima da extensão considerada ideal para artigos originais. Sugerimos especial atenção aos seguintes pontos: (a) resumir a discussão sobre as vacinas mais prescritas, evitando trechos com foco excessivamente clínico ou descritivo; (b) eliminar trechos redundantes que retomam conceitos já explorados (ex.: papel dos CRIE, descentralização, equidade); (c) priorizar evidências que dialoguem diretamente com o delineamento e escopo do estudo, evitando revisões amplas sem conexão com os resultados apresentados.

3. Solicitamos a padronização do alinhamento do conteúdo das tabelas, conforme as normas editoriais da RESS: os textos (nomes das variáveis, categorias ou descrições) devem estar alinhados à esquerda nas células correspondentes; os dados numéricos (frequências, percentuais, medidas de associação, intervalos de confiança) devem estar alinhados à direita.

4. Tabela 2: remover os totais (n = xx) indicados ao lado dos rótulos “Sexo” e “Faixa etária (anos)”, uma vez que essa informação já está apresentada nas linhas correspondentes da tabela. A repetição no cabeçalho pode comprometer a clareza visual e contraria a padronização adotada pela RESS. Como alternativa editorialmente adequada, sugerimos incluir uma nota de rodapé explicando que os totais variam devido à presença de dados ausentes, por exemplo: “Nota: os totais por variável podem variar em razão de registros incompletos.”

Solicito que todos os comentários da revisão por pares sejam respondidos pontualmente por meio de uma carta resposta detalhada, explicando as modificações realizadas ou justificando, quando necessário, a manutenção do texto original.

A nova versão do manuscrito será reconsiderada após essa reformulação e poderá passar por nova rodada de avaliação.

Recommendation: Minor revision

## Third round comments

Prezados,

Agradecemos pelo envio da nova versão do manuscrito e pelas informações inseridas na seção “Disponibilidade dos dados”. O trecho fornecido do parecer do CEP reforça a confidencialidade no manuseio dos dados durante a coleta e análise, mas não apresenta vedação explícita quanto à possibilidade de disponibilização pública de uma versão anonimizada do banco de dados para fins científicos.

Assim, permanecemos com as seguintes dúvidas:

- Foi considerada a possibilidade de compartilhamento de uma versão anonimizada do banco de dados?

- Em caso negativo, por que isso não é possível? Existe orientação expressa no TCLE ou no parecer do CEP que proíba essa divulgação, mesmo com anonimização?

Reforçamos que, caso os dados não possam ser compartilhados, a seção “Disponibilidade dos dados” do manuscrito deve apresentar uma justificativa objetiva e completa, informando que a decisão decorre de impedimentos éticos definidos pelo comitê de ética, e não apenas de decisões operacionais da equipe de pesquisa.

Aguardamos esse esclarecimento para concluir a avaliação editorial.

Recommendation: Minor revision

